# Potential Antitumor Effects of 6-Gingerol in p53-Dependent Mitochondrial Apoptosis and Inhibition of Tumor Sphere Formation in Breast Cancer Cells

**DOI:** 10.3390/ijms22094660

**Published:** 2021-04-28

**Authors:** Nipin Sp, Dong Young Kang, Jin-Moo Lee, Se Won Bae, Kyoung-Jin Jang

**Affiliations:** 1 Department of Pathology, School of Medicine, Institute of Biomedical Science and Technology, Konkuk University, Chungju 27478, Korea; nipinsp@konkuk.ac.kr (N.S.); kdy6459@kku.ac.kr (D.Y.K.); 2 Pharmacological Research Division, National Institute of Food and Drug Safety Evaluation, Osong Health Technology Administration Complex, Cheongju 28159, Korea; elzem@korea.kr; 3 Department of Chemistry and Cosmetics, Jeju National University, Jeju 63243, Korea; swbae@jejunu.ac.kr

**Keywords:** 6-Gingerol, DDR, G0/G1 arrest, mitochondrial apoptosis, tumorsphere, p53, EGFR/Src/STAT3

## Abstract

Hormone-specific anticancer drugs for breast cancer treatment can cause serious side effects. Thus, treatment with natural compounds has been considered a better approach as this minimizes side effects and has multiple targets. 6-Gingerol is an active polyphenol in ginger with various modalities, including anticancer activity, although its mechanism of action remains unknown. Increases in the level of reactive oxygen species (ROS) can lead to DNA damage and the induction of DNA damage response (DDR) mechanism, leading to cell cycle arrest apoptosis and tumorsphere suppression. Epidermal growth factor receptor (EGFR) promotes tumor growth by stimulating signaling of downstream targets that in turn activates tumor protein 53 (p53) to promote apoptosis. Here we assessed the effect of 6-gingerol treatment on MDA-MB-231 and MCF-7 breast cancer cell lines. 6-Gingerol induced cellular and mitochondrial ROS that elevated DDR through ataxia-telangiectasia mutated and p53 activation. 6-Gingerol also induced G0/G1 cell cycle arrest and mitochondrial apoptosis by mediating the BAX/BCL-2 ratio and release of cytochrome c. It also exhibited a suppression ability of tumorsphere formation in breast cancer cells. EGFR/Src/STAT3 signaling was also determined to be responsible for p53 activation and that 6-gingerol induced p53-dependent intrinsic apoptosis in breast cancer cells. Therefore, 6-gingerol may be used as a candidate drug against hormone-dependent breast cancer cells.

## 1. Introduction

Globally, breast cancer has one of the highest mortality rates of cancer among women, and it has been identified as the fifth most common cancer type in Korea, accounting for more than 20% of cancers [1]. The incidence of breast cancer has increased significantly since 1970, which can be linked to modern living, particularly as environmental factors are known to play a key role in tumorigenesis [2]. Only 5–10% of breast cancer cases are caused by genetic inheritance of the breast cancer 1/2 (BRCA1/2) gene mutations [3]. Estrogen and progesterone receptors (ER and PR) and human epidermal growth factor 2 (HER2) statuses also play a vital role in breast cancer development [4].

Hormone receptor status is highly useful in breast cancer treatment; however, chemotherapy is limited by possible adverse effects. Natural compounds for anticancer treatment can be effectively assessed for activity using hormone-specific breast cancer cells such as MCF-7 (ER-α positive) or MDA-MB-231 (triple negative) [5]. A number of natural compounds have been found to have anticancer properties against different types of cancer without significant side effects [6,7,8]. Ginger is a popular spice widely used in Asia, with 6-gingerol identified as one of its bioactive phenolic compounds [9]. 6-Gingerol is considered as the main pharmacological component of ginger as it has anti-oxidant, anti-platelet, anti-inflammatory, anti-proliferative, and anti-cancer activities [10,11,12,13,14,15]. 6-Gingerol has been reported to induce antitumor activity against various types of cancers such as pancreatic [16], gastric [17], colorectal [18], and renal [19]. However, the exact mechanism of cell death by 6-gingerol in breast cancer is yet to be discovered.

Reactive oxygen species (ROS) have been determined to have a critical role in cancer as they induce DNA damage that leads to DNA damage response (DDR) mechanism. Generally, prolonged stress leads to the induction of ROS at both cellular and mitochondrial levels, resulting in double-strand DNA breaks and loss of heterozygosity or genomic instability. In response to this phenomenon, the DDR activates DNA repair against aberrant DNA [20,21]. The molecular mechanism of DDR begins with ataxia-telangiectasia mutated (ATM), ATM- and Rad3-related (ATR), and DNA-dependent protein kinase (DNA-PKcs) [22,23]. ATM signaling activates the expression of tumor protein 53 (p53) in response to DNA damage in order to induce DDR, cell cycle arrest, and apoptosis mechanisms [24]. In breast cancer cells, p53 activates ATM phosphorylation, which further results in G2/M cell cycle arrest and apoptosis in MDA-MB-231 cells [25]. An anticancer drug that induced ROS formation could result in increasing DNA damage and activation of the DDR as a result of increasing expression of the ATM/ATR signaling pathway.

p53 has been determined to be a nuclear transcription factor that is a commonly mutated gene in cancer especially in MDA-MB-231 triple-negative breast cancer cells; however, p53 also plays a vital role in apoptosis induction where mutated p53 is stabilized by elevated levels of phospholipase D activity [26] or via activation of p73 and p53 upregulated modulator of apoptosis (PUMA) [27]. p53-dependent cell cycle arrest and apoptosis in MDA-MB-231 cells were also demonstrated by a bioactive component ziyuglycoside I, a major extracted component of the herb *Radix Sanguisorbae* [28]. p53 has been considered as a tumor suppressor gene because of the promotion of cell cycle arrest in cancer cells by the activation of another tumor suppressor protein 21 (p21) and inhibition of cyclin-dependent kinase proteins that enable the induction of programmed cell death. Moreover, p53 is a major factor in apoptosis, and loss of apoptosis has been correlated with tumor development in p53-null transgenic mice, indicating the tumor suppressor ability of p53 [29]. RING-finger type E3 ubiquitin protein ligase MDM2 is a negative regulator of p53 and enables the degradation of p53 through the proteasome, whereas conditions of stress lead to blockage of the p53-MDM2 interaction, resulting in p53 stabilization. Hence, the p53-MDM2 interaction is a major component of tumorigenesis as well as tumorsphere formation [30,31].

Epidermal growth factor receptor (EGFR) promotes tumor growth by activating proto-oncogenes following phosphorylation. Elevated levels of EGFR were observed in various human cancers, which enabled the signal transduction in cell cytoplasm and thereby promote tumorigenesis. EGFR can contribute to transformation of cellular phenotypes that enable tumor cell growth and survival [32,33]. EGFR signaling can also induce immune activation by activating p53 signaling, which thereby inhibits PD-L1 expression [34]. Steroid receptor coactivator (Src) is an intracellular tyrosine kinase that takes part in tumorigenesis via signaling from EGFR through association with focal adhesion kinase (FAK) [35]. Src plays a major role in cancer cell growth and survival, and thereby tumor angiogenesis, by acting as an initial prompt in molecular signaling pathways that transmit signals from EGFR to their downstream targets to promote tumor invasion and metastasis [36,37]. Signal transducer and activator of transcription 3 (STAT3) is a well-known oncogene that acts as a potential regulator for EGFR-mediated apoptosis, and phosphorylation of STAT3 has been determined to induce STAT3 dimerization via Src homology 2 (SH2) domain interactions to direct the translocation of STAT3 to the nucleus and initiate gene transcription [38]. In the nucleus, activated STAT3 binds to the p53 promoter to inhibit p53 endogenous protein expression, and blockage of STAT3 leads to induction of p53 expression [39]. EGFR-Src-STAT3 signaling is also considered as an important pathway in tumorigenesis in various cancer types [40,41]. Therefore, EGFR/Src/STAT3 signaling through p53 is important in the induction of DDR, cell cycle arrest, and apoptosis in cancer cells.

In this study, we aimed to examine the ability of 6-gingerol to induce ROS, DDR, cell cycle arrest, and apoptosis in breast cancer cells. We also aimed to discover the molecular mechanism underlying p53-dependent intrinsic apoptosis by 6-gingerol.

## 2. Results

### 2.1. 6-Gingerol Inhibits Cell Proliferation and Induces ROS in Breast Cancer Cells

**Figure 1:**
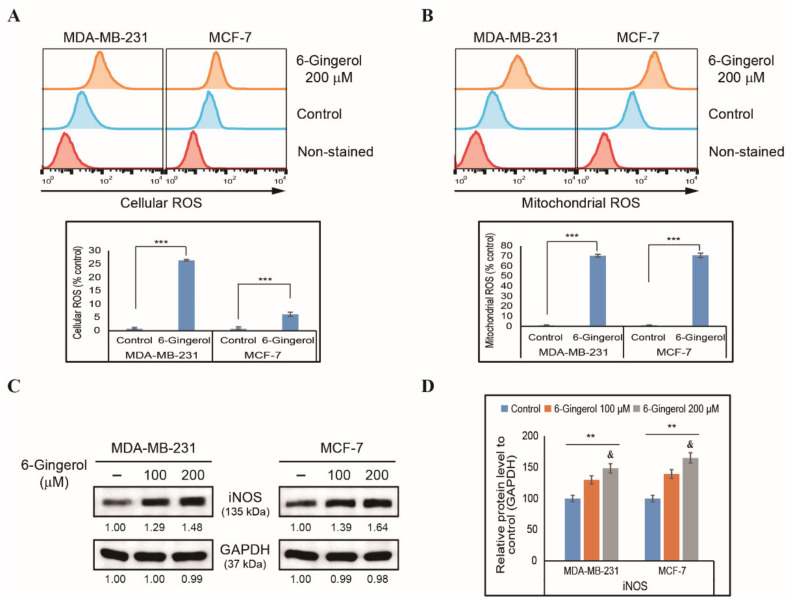
Induction of ROS in breast cancer cells by 6-gingerol. (**A**) Flow cytometry analysis of cellular ROS in MDA-MB-231 and MCF-7 cells after treatment with 6-gingerol for 48 h. Graphical representation shows the percentage of cells with ROS induction. Values are presented as mean ± SEM of three independent experiments performed in triplicate (n = 3). *** *p* < 0.001 (Student’s *t*-test). (**B**) Flow cytometry analysis of mitochondrial ROS by 6-gingerol in MDA-MB-231 and MCF-7 cells for 48 h. Graphical representation shows the percentage of cells with mitochondrial ROS. The values are presented as mean ± SEM of three independent experiments performed in triplicate (n = 3). *** *p* < 0.001 (Student’s *t*-test). (**C**) Western blotting of MDA-MB-231 and MCF-7 cells with 100 and 200 µM 6-gingerol for 48 h showing the expression of iNOS. (**D**) The relative expressions of iNOS proteins were determined via densitometry and normalized to GAPDH. Controls are set to 100. Data were confirmed after repeating the experiment three times. ** *p* < 0.01 (ANOVA test) and *p* < 0.01 vs. control.

To elucidate the effect of 6-gingerol on cancer cells, we initially assessed cell proliferation in MDA-MB-231 and MCF-7 breast cancer cells and identified a concentration-dependent cell proliferation inhibition by 6-gingerol when tested for 48 h (Figure S1A). The approximate IC_50_ dosage was 200 µM; therefore, we used 6-gingerol at 100 and 200 µM for further studies. In order to analyze the toxicity of 6-gingerol in normal cells, we used human breast epithelial cell, MCF-10A and analyzed cell viability with increasing concentration of 6-gingerol for 48 h and observed a non-significant cell viability inhibition even up to a concentration of 500 µM 6-gingerol (Figure S1B). As cell morphology may change considerably during cell division or cell death, we also checked the morphology of MDA-MB-231 and MCF-7 cells after treatment with 6-gingerol for 48 h. Inverted phase-contrast microscopy showed nuclear abnormalities after 6-gingerol treatment and fragmentation of nuclei, which indicated the possible induction of apoptosis (Figure S1C). 6-Gingerol can induce cell death and nuclear abnormalities in breast cancer cells, and we therefore analyzed whether ROS was also induced, as this is crucial in anticancer processes. We found that 200 µM of 6-gingerol can significantly induce cellular ROS levels in both MDA-MB-231 and MCF-7 cells (Figure 1A). This increase of ROS levels by 6-gingerol may indicate ROS induction in mitochondria, as the mitochondria are the main source of cellular ROS. Flow cytometry analysis of mitochondrial ROS also demonstrated a similar increase in ROS levels following treatment with 200 µM of 6-gingerol in both breast cancer cell lines (Figure 1B). ROS production can be affected by inducible nitric oxide synthase (iNOS), and we therefore checked the expression levels of iNOS in MDA-MB-231 and MCF-7 cells after treatment with 100 and 200 µM 6-gingerol by Western blotting (Figure 1C). Based on our findings, there was a significant increase in the expression levels of iNOS, which clearly indicated the role of 6-gingerol in the induction of ROS (Figure 1D). Altogether, these results suggest that 6-gingerol may have anticancer activity against breast cancer cells.

### 2.2. 6-Gingerol Induces DDR in Breast Cancer Cells

**Figure 2:**
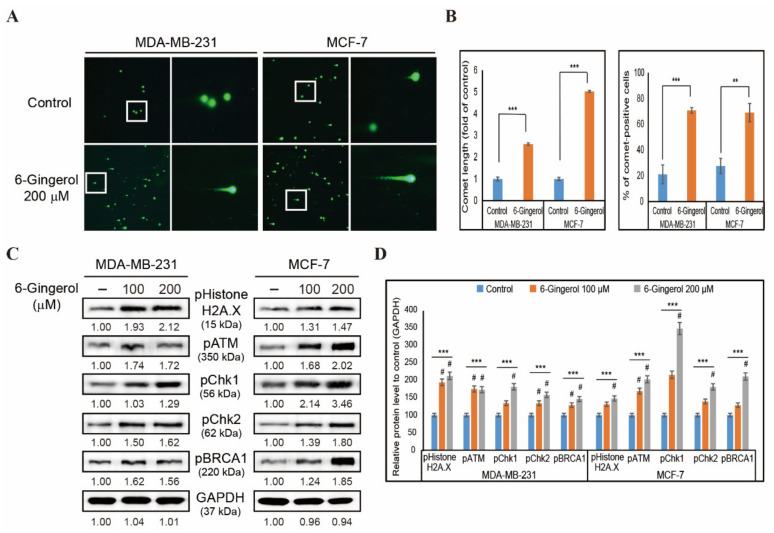
6-Gingerol induces DDR in breast cancer cells. (**A**) Comet assay images from fluorescent microscopy analysis at 10× and 40× magnification showing fragmented DNA migration from the nucleoid body that forms a comet tail in MDA-MB-231 and MCF-7 cells after 48 h treatment with 6-gingerol. (**B**) Graphical representation of comet length was analyzed as the fold change versus the control and % of comet-positive cells with 6-gingerol treatment in breast cancer cells. ** *p* < 0.01 and *** *p* < 0.001 (Student’s *t*-test). (**C**) Western blotting of MDA-MB-231 and MCF-7 cells treated for 48 h with 100 and 200 µM of 6-gingerol showing the expression of phospho-histone H2A.X, ATM, CHK1, CHK2, and BRCA1. (**D**) Representative expressions of proteins were determined by densitometry and normalized to GAPDH. Controls are set to 100. Data were confirmed after repeating the experiment three times. *** *p* < 0.001 (ANOVA test). # *p* < 0.001 vs. control.

The induction of cellular ROS as well as mitochondrial ROS by 6-gingerol indicated that 6-gingerol may also induce DDR in breast cancer cells. In order to verify this, we used a comet assay in MDA-MB-231 and MCF-7 cells with 6-gingerol treatment for 48 h. Fluorescent microscopy images of comet formation by 200 µM of 6-gingerol in both MDA-MB-231 and MCF-7 cells indicated the induction of DNA double strand breaks in cancer cells (Figure 2A). There was a significant increase in comet length and comet-positive cells following treatment with 6-gingerol compared with those in non-treated control cells, suggesting that 6-gingerol may induce DDR (Figure 2B). To confirm induction of DDR by 6-gingerol, we assessed MDA-MB-231 and MCF-7 cells by Western blotting to determine the proteins responsible for DDR (Figure 2C). Based on our findings, there was a significant increase in the expression levels of phosphorylated histone H2A.X, ATM, CHK1, CHK2, and BRCA1 in both breast cancer cell lines (Figure 2D) which suggested that ATM acts as the key regulator in 6-gingerol-induced DDR.

### 2.3. 6-Gingerol Induces G0/G1 Cell Cycle Arrest in Breast Cancer Cells

**Figure 3:**
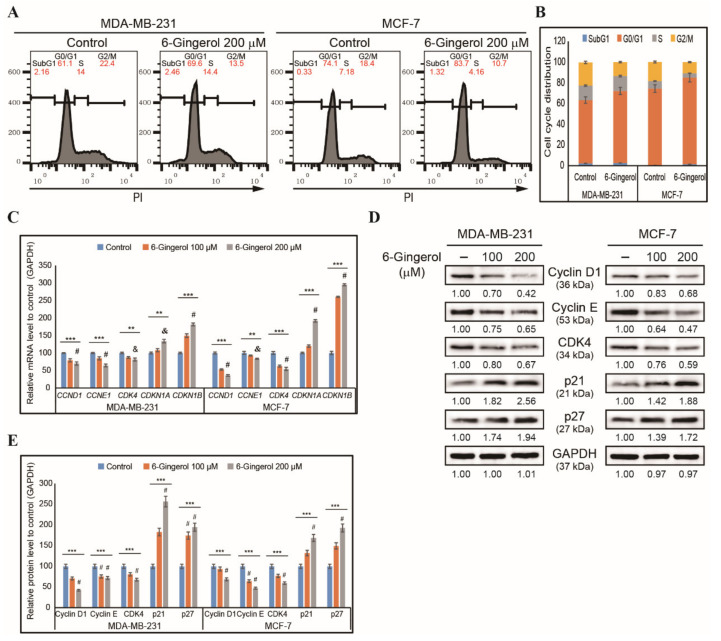
6-Gingerol induces cell cycle arrest. (**A**) Flow cytometry analysis using PI staining showing cell cycle distribution in MDA-MB-231 and MCF-7 cells after 48 h treatment with 6-gingerol. (**B**) Graphical representation of cell cycle distribution showing G0/G1 arrest by 6-gingerol in breast cancer cells. (**C**) RT-qPCR showing expression of cell cycle checkpoint genes. The representative expressions of *CCND1*, *CCNE1*, *CDK4*, *CDKN1A*, and *CDKN1B* mRNA are shown; Cp values were normalized to *GAPDH* mRNA. Controls are set to 100. ** *p* < 0.01 and *** *p* < 0.001 (ANOVA test), & *p* < 0.01 vs. control, and # *p* < 0.001 vs. control. (**D**) Western blotting of MDA-MB-231 and MCF-7 cells after 48 h treatment with 100 and 200 µM of 6-gingerol showing the expression of cyclin D1, cyclin E, CDK4, p21, and p27. (**E**) Representative protein expression was determined by densitometry and normalized to GAPDH. Controls are set to 100. Data were confirmed after repeating the experiment three times. *** *p* < 0.001 (ANOVA test). # *p* < 0.001 vs. control.

Based on our findings, we determined that 6-gingerol could induce DDR in breast cancer cells, suggesting that it could leads to cell cycle arrest. Therefore, we checked the cell cycle distribution of MDA-MB-231 and MCF-7 cells after 48 h treatment with 200 µM of 6-gingerol using flow cytometry with PI staining (Figure 3A). Our results suggested an arrest in the G0/G1 phase of the cell cycle by 6-gingerol, which indicates that DNA damage triggers to DDR which upon failure culminates in prolonged cell cycle arrest in breast cancer cells (Figure 3B). For confirmation, we used RT-qPCR to analyze the following cell cycle check point genes, *CCND1*, *CCNE1*, *CDK4*, *CDKN1A*, and *CDKN1B* in MDA-MB-231 and MCF-7 cells after 48 h treatment with 200 µM of 6-gingerol. This demonstrated a downregulation of the expression of *CCND1*, *CCNE1*, and *CDK4* mRNA and an upregulation of expression of tumor suppressor genes *CDKN1A* and *CDKN1B* (Figure 3C). The cell cycle arrest ability of 6-gingerol was confirmed by analyzing the cell cycle regulator proteins cyclin D1, cyclin E, CDK4, p21, and p27 by Western blotting (Figure 3D). There was a similarly significant decrease in the expression of cyclin D1, cyclin E, and CDK4 proteins and an increase in the expression of p21 and p27 proteins (Figure 3E). Thus, induction of DDR and cell cycle arrest suggest the possibility of apoptosis induction by 6-gingerol in breast cancer cells.

### 2.4. 6-Gingerol Induces Intrinsic Apoptosis in Breast Cancer Cells

**Figure 4:**
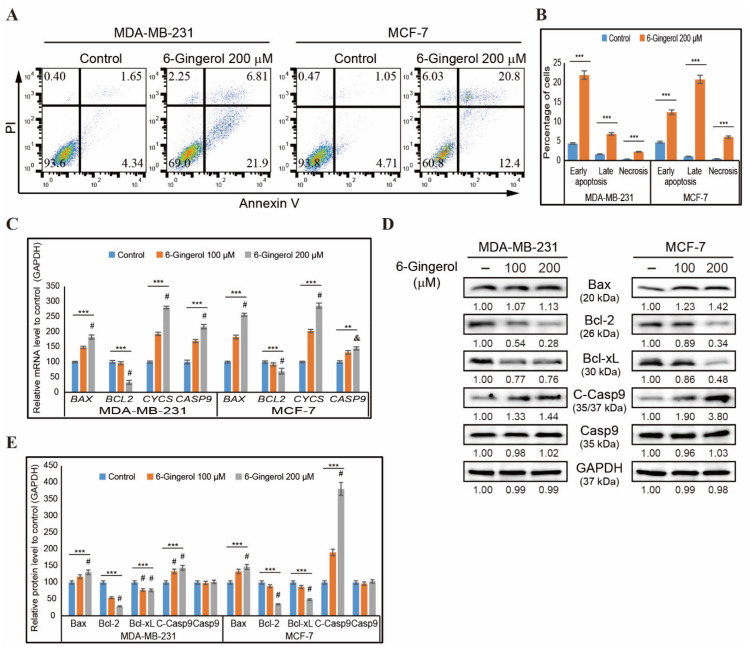
6-Gingerol induces apoptosis. (**A**) Flow cytometry analysis using annexin V and PI staining in MDA-MB-231 and MCF-7 cells after treatment with 6-gingerol for 48 h. (**B**) Graphical representation of the percentage of cells undergoing phases of apoptosis after treatment with 200 µM of 6-gingerol in breast cancer cells. The values are presented as means ± SEM of three independent experiments performed in triplicate (n = 3). *** *p* < 0.001 (Student’s *t*-test). (**C**) qPCR analysis of expression of intrinsic apoptosis genes. The representative expression of the *BAX*, *BCL-2*, *CYCS*, and *CASP9* mRNA is shown; Cp values were then normalized to *GAPDH* mRNA. Controls are set to 100. ** *p* < 0.01 and *** *p* < 0.001 (ANOVA test), and & *p* < 0.01 vs. control. # *p* < 0.001 vs. control. (**D**) Western blotting analysis of MDA-MB-231 and MCF-7 cells after 48 h treatment with 100 and 200 µM 6-gingerol showing the expression of BAX, BCL-2, BCL-xL, C-caspase 9, and caspase 9. (**E**) The representative expression of proteins was determined by densitometry and normalized to GAPDH. Controls are set to 100. Data were confirmed after repeating the experiment three times. *** *p* < 0.001 (ANOVA test). # *p* < 0.001 vs. control.

The induction of DDR and cell cycle arrest suggested an apoptosis induction by 6-gingerol. We then used flow cytometry to assess whether 6-gingerol induced apoptosis in MDA-MB-231 and MCF-7 cells (Figure 4A). Based on our results, 6-gingerol induced earlier apoptosis in a greater percentage of MDA-MB-231 cells compared with the later induction of apoptosis seen in MCF-7 cells (Figure 4B). After confirming apoptosis induction, we checked the type of apoptosis induced. We used RT-qPCR to analyze mRNA expression of the genes responsible for the intrinsic apoptosis pathway, including *BAX*, *BCL-2*, *CYCS*, and *CASP9* and found an increase in the expressions of *BAX*, *CYCS*, and *CASP9* and a decrease in the expression of *BCL-2* (Figure 4C). Then, we confirmed the changes in expression of the intrinsic pathway of apoptosis at the translational level by Western blotting in MDA-MB-231 and MCF-7 cells after 48 h treatment with 6-gingerol (Figure 4D). These results also showed similar results of upregulated expression of BAX, cleaved caspase 9, and cytochrome c proteins and downregulated expression of BCL-2 and BCL-xL proteins with unchanged levels of total caspase 9 with 100 and 200 µM of 6-gingerol treatment (Figure 4E). We have also confirmed 6-gingerol induction of the intrinsic pathway of apoptosis via analyses of the BAX/BCL-2 ratio at the mRNA and protein levels and demonstrated a significant increase in these ratios (Figure S2A).

### 2.5. 6-Gingerol Induces Loss of Mitochondrial Membrane Potential and Release of Cytochrome c

**Figure 5:**
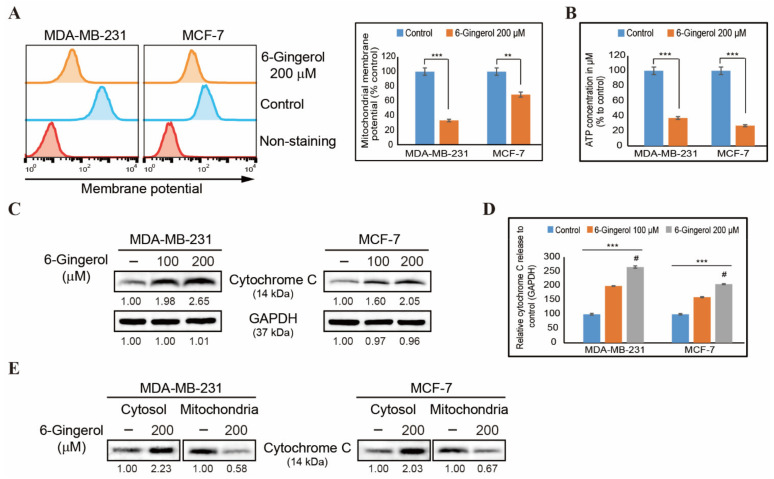
6-Gingerol induces the release of cytochrome c. (**A**) Flow cytometry analysis in MDA-MB-231 and MCF-7 cells after treatment with 6-gingerol for 48 h showing mitochondrial membrane potential and the resulting decrease in membrane potential. The values are presented as means ± SEM of three independent experiments performed in triplicate (n = 3). ** *p* < 0.01 and *** *p* < 0.001 (Student’s *t*-test). (**B**) Graphical representation of ATP formation induced by treatment with 200 µM of 6-gingerol in breast cancer cells. The values are presented as means ± SEM of three independent experiments performed in triplicate (n = 3). *** *p* < 0.001 (Student’s *t*-test). (**C**) Western blotting of MDA-MB-231 and MCF-7 cells after 48 h treatment with 100 and 200 µM of 6-gingerol showing the expression of cytochrome c. (**D**) Representative expression of proteins was determined by densitometry and normalized to GAPDH. Controls are set to 100. Data were confirmed after repeating the experiment three times. *** *p* < 0.001 (ANOVA test). # *p* < 0.001 vs. control. (**E**) Western blotting analysis in cytosolic and mitochondrial fractions isolated from MDA-MB-231 and MCF-7 cells after 48 h treatment with 200 µM of 6-gingerol showing the expression of cytochrome c protein.

As 6-gingerol induced the intrinsic pathway of apoptosis, we next evaluated the role of mitochondria during apoptosis. First, treatment of MDA-MB-231 and MCF-7 cells with 6-gingerol resulted in loss of mitochondrial membrane potential (Figure 5A). This indicated a loss of integrity in the mitochondria membrane due to an increase in the expression of BAX and a decrease in the expression of BCL-2. We also observed a similar downregulation in ATP formation in the two breast cancer cell lines following 6-gingerol treatment, which suggested the loss of mitochondrial activity due to pore formation to proceed for mitochondrial apoptosis (Figure 5B). The release of BAX and inhibition of BCL-2 indicate that pores were formed in the mitochondrial membrane to enable the release of cytochrome c to signal intrinsic apoptosis through caspase 9 activation. Levels of cytochrome c protein in MDA-MB-231 and MCF-7 cells were significantly elevated following 6-gingerol treatment (Figure 5C,D), indicative of the release of cytochrome c induced by 6-gingerol. To confirm the release of cytochrome c from mitochondria to the cytosol, we isolated the cytosolic and mitochondrial fractions from breast cancer cells and analyzed the protein expression levels of cytochrome c by Western blotting (Figure 5E). This demonstrated a decrease in the levels of cytochrome c in the mitochondria and an increase in the levels of cytochrome c in the cytosol. Thus, cytochrome c is released as a result of the increase in the BAX/BCL-2 ratio following 6-gingerol treatment and further stimulates mitochondrial intrinsic apoptosis through activation of caspase 9.

### 2.6. 6-Gingerol Inhibits EGFR/Src/STAT3 Pathway and Signals to p53 Upregulation

**Figure 6:**
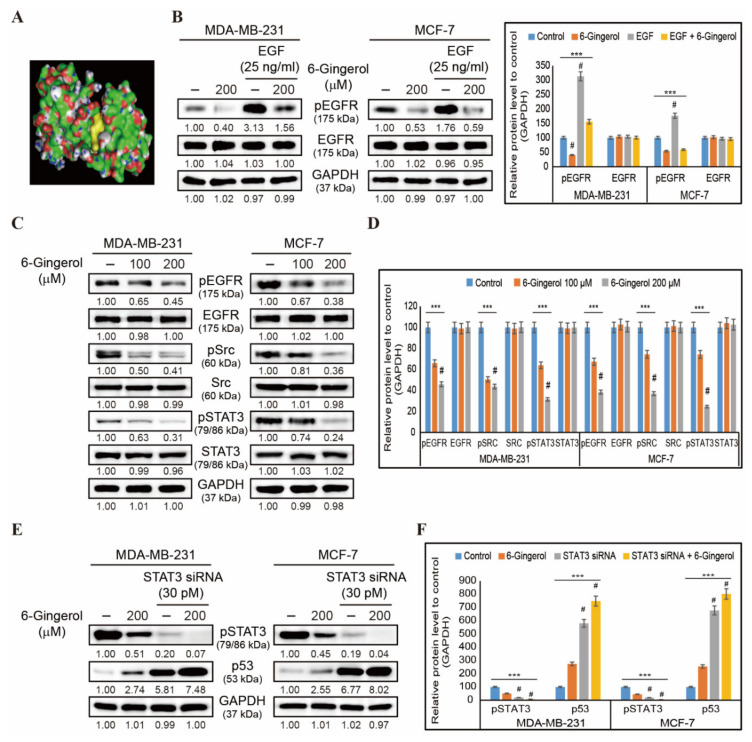
6-Gingerol regulates EGFR/Src/STAT3 and p53 signaling. (**A**) Molecular docking using AutoDock Vina software that shows the binding of 6-gingerol (PubChem ID: 442793) to the ATP-binding domain of EGFR (PDB ID: 2GS2). (**B**) Western blotting of MDA-MB-231 and MCF-7 cells pre-treated with recombinant EGF (25 ng/mL) for 1 h and then treated with 200 µM 6-gingerol for 48 h showing the expression patterns of phosphorylated EGFR and total EGFR proteins. Representative expression of proteins was determined by densitometry and normalized to GAPDH. Controls are set to 100. Data were confirmed after repeating the experiment three times. *** *p* < 0.001 (ANOVA test). # *p* < 0.001 vs. control. (**C**) Western blotting of MDA-MB-231 and MCF-7 cells following 48 h treatment with 100 and 200 µM of 6-gingerol showing expression levels for pEGFR, EGFR, pSrc, Src, pSTAT3, and STAT3. (**D**) Representative expression of proteins was determined by densitometry and normalized to GAPDH. Controls are set to 100. Data were confirmed after repeating the experiment three times. *** *p* < 0.001 (ANOVA test). # *p* < 0.001 vs. control. (**E**) Western blotting of MDA-MB-231 and MCF-7 cells pre-treated with STAT3 siRNA (30 pM) for 24 h and then treated with 200 µM 6-gingerol for 48 h showing the expression patterns of phosphorylated STAT3 and p53 proteins. (**F**) Representative expression of proteins was determined by densitometry and normalized to GAPDH. Controls are set to 100. Data were confirmed after repeating the experiment three times. *** *p* < 0.001 (ANOVA test). # *p* < 0.001 vs. control.

Our initial results demonstrated that 6-gingerol induced mitochondrial apoptosis in breast cancer cells. We then analyzed the molecular signaling behind this mechanism. First, we analyzed the binding of 6-gingerol to EGFR using molecular docking wherein we were able to determine a strong binding of 6-gingerol with the EGF receptor with a binding affinity of −6.2 kcal/mol, indicating that 6-gingerol acts through EGFR signaling (Figure 6A). In order to confirm the binding of 6-gingerol to EGF receptor, we treated the breast cancer cells with recombinant human EGF (25 ng/mL) which showed an increase in the expression of phosphorylated EGFR which significantly downregulated by 6-gingerol (Figure 6B). To analyze the downstream targets of EGFR signaling, we checked the expression of pEGFR, EGFR, pSrc, Src, pSTAT3, and STAT3 via Western blotting in MDA-MB-231 and MCF-7 cells following treatment with 100 and 200 µM of 6-gingerol for 48 h (Figure 6C). This demonstrated a significant concentration-dependent inhibition of EGFR/Src/STAT3 signaling by 6-gingerol in MDA-MB-231 and MCF-7 cells (Figure 6D). Signaling through this pathway could lead to blockage of p53-MDM2 interaction. Hence, we analyzed the relationship between STAT3 and p53 in presence of 6-gingerol using STAT3 gene silencing by siRNA (Figure 6E). Obtained results showed a significant inhibition in phospho-STAT3 expression in STAT3 siRNA treated cells which further inhibited by 6-gingerol, whereas the STAT3 silencing resulted in p53 upregulation and addition of 6-gingerol significantly increased the expression of p53 furthermore (Figure 6F). These results suggested the ability of 6-gingerol to induce p53 expression through regulating EGFR/Src/STAT3 signaling.

### 2.7. 6-Gingerol Induces p53-Dependent Intrinsic Apoptosis in Breast Cancer Cells

**Figure 7:**
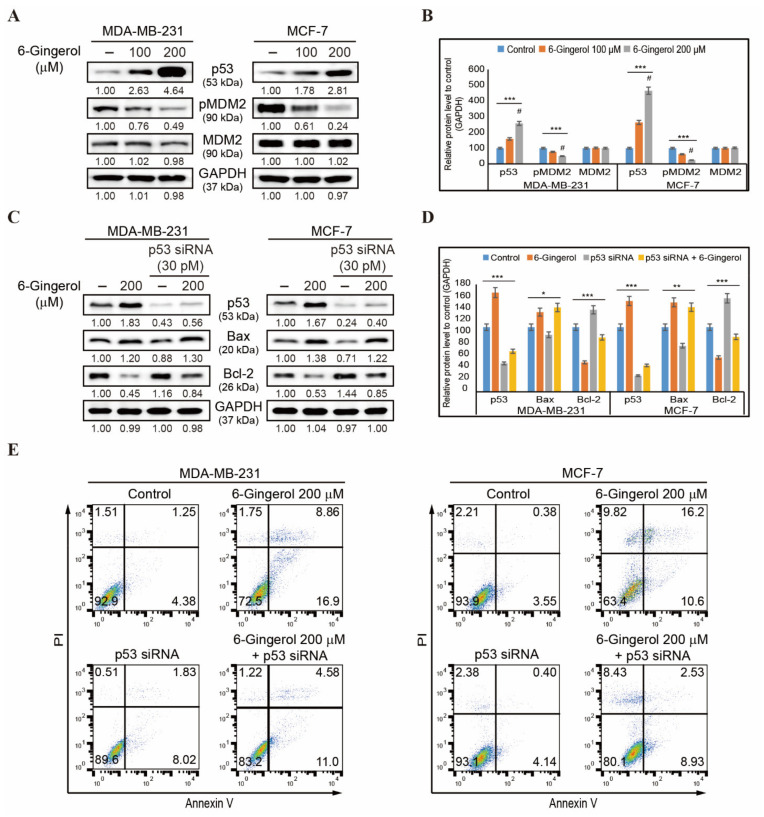
6-Gingerol induces p53-dependent apoptosis. (**A**) Western blotting of total proteins isolated from MDA-MB-231 and MCF-7 cells after 48 h treatment with 100 and 200 µM of 6-gingerol showed the expression of p53, pMDM2, and MDM2 proteins. (**B**) Representative expression of proteins was determined by densitometry and normalized to GAPDH. Controls are set to 100. Data were confirmed after repeating the experiment three times. *** *p* < 0.001 (ANOVA test). # *p* < 0.001 vs. control. (**C**) Western blotting of total proteins isolated from MDA-MB-231 and MCF-7 cells after 48 h treatment with 200 µM of 6-gingerol, p53 siRNA, and p53 siRNA + 6-gingerol showing the expression of p53, BAX, and BCL-2 proteins. (**D**) Representative expression of proteins was determined by densitometry and normalized to GAPDH. Controls are set to 100. Data were confirmed after repeating the experiment three times. * *p* < 0.05, ** *p* < 0.01, and *** *p* < 0.001 (ANOVA test). (**E**) Flow cytometry analysis using annexin V and PI staining in MDA-MB-231 and MCF-7 cells after treatment with 6-gingerol, p53 siRNA, and p53 siRNA + 6-gingerol for 48 h.

Our results demonstrated that STAT3 signaling plays an important role in p53 induction by 6-gingerol. We then analyzed p53-MDM2 interaction in breast cancer cells by 6-gingerol. The expression of p53, pMDM2, and MDM2 proteins were detected by Western blotting (Figure 7A) wherein we found an increase in the expression of p53 and a decrease in the expression of phospho-MDM2 in both cell lines (Figure 7B). Total MDM2 remained unchanged after treatment with 100 and 200 µM of 6-gingerol. Hence, we suggest that the 6-gingerol inhibition of EGFR/Src/STAT3 pathway regulates the increase in p53 expression and that this then mediates changes to expression of BAX and BCL-2 to induce the intrinsic apoptosis pathway. Our results also demonstrated that p53 plays an important role in apoptosis induced by 6-gingerol. However, MDA-MB-231 is known as a p53-mutated cell line. Therefore, to prove the role of p53 in molecular signaling of apoptosis, we silenced p53 expression with siRNA and analyzed the expression levels of BAX and BCL-2 (Figure 7C). This demonstrated a decrease in the expression of BAX in p53 siRNA-treated cells compared in 6-gingerol-treated cells, whereas a combination of p53 siRNA and 6-gingerol increased the expression levels of BAX in comparison with those in p53 siRNA-treated cells. The expression of BCL-2 was the opposite to that of BAX expression, which clearly suggested that there was a p53-dependent intrinsic apoptosis induction by 6-gingerol (Figure 7D). The BAX/BCL-2 ratio was elevated in the presence of p53 siRNA when treated in combination with 6-gingerol compared with that found with treatment of cells with p53 siRNA only (Figure S2B). These results strongly suggested the active participation of p53 in the mitochondrial apoptosis induction by 6-gingerol in breast cancer cells. To confirm the role of p53 in apoptosis, we used gene silencing by p53 siRNA and analyzed apoptosis by flow cytometry (Figure 7E). This demonstrated a decrease in apoptosis following p53 siRNA treatment alone in comparison with treatment with 6-gingerol. Treatment with 6-gingerol along with p53 siRNA was determined to increase the rate of apoptosis, highlighting the role of p53 in apoptosis induction by 6-gingerol.

### 2.8. 6-Gingerol Inhibits p53-Dependent Tumorsphere Formation in Breast Cancer Cells

**Figure 8:**
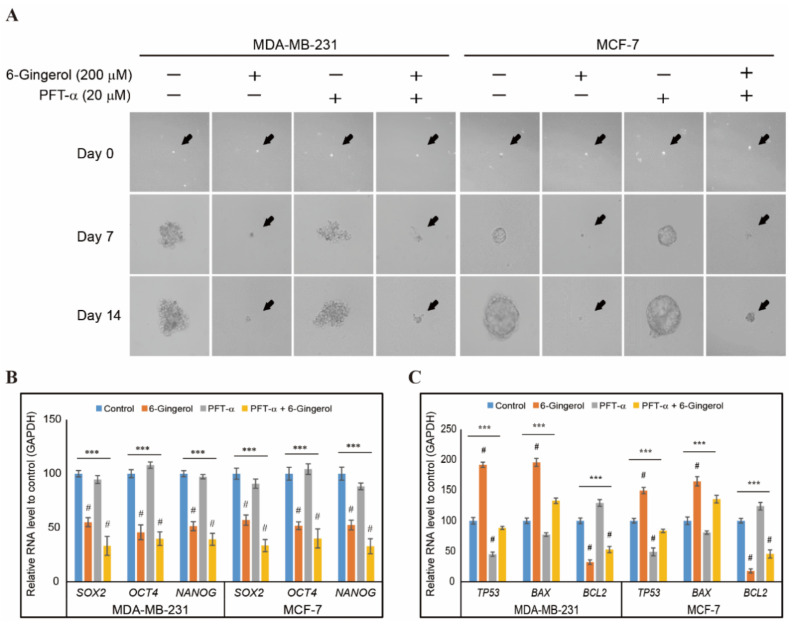
6-gingerol inhibits tumorsphere formation. (**A**) Suppression in sphere forming ability of MDA-MB-231 and MCF-7 cells by 6-gingerol treatment. Representative photographs are presented (scale bar, 15 μm). Cells were incubated with sphere forming media for 14 days with or without 200 µM 6-gingerol and 20 µM PFT-α. (**B**) qPCR analysis of expression of cancer stem cell marker genes. The representative expression of the *SOX2, OCT4,* and *NANOG* mRNA is shown; Cp values were then normalized to *GAPDH* mRNA. Controls are set to 100. *** *p* < 0.001 (ANOVA test). # *p* < 0.001 vs. control. (**C**) qPCR analysis of expression of p53 and intrinsic apoptosis genes. The representative expression of the *TP53, BAX,* and *BCL-2* mRNA is shown; Cp values were then normalized to *GAPDH* mRNA. Controls are set to 100. *** *p* < 0.001 (ANOVA test). # *p* < 0.001 vs. control.

**Figure 9:**
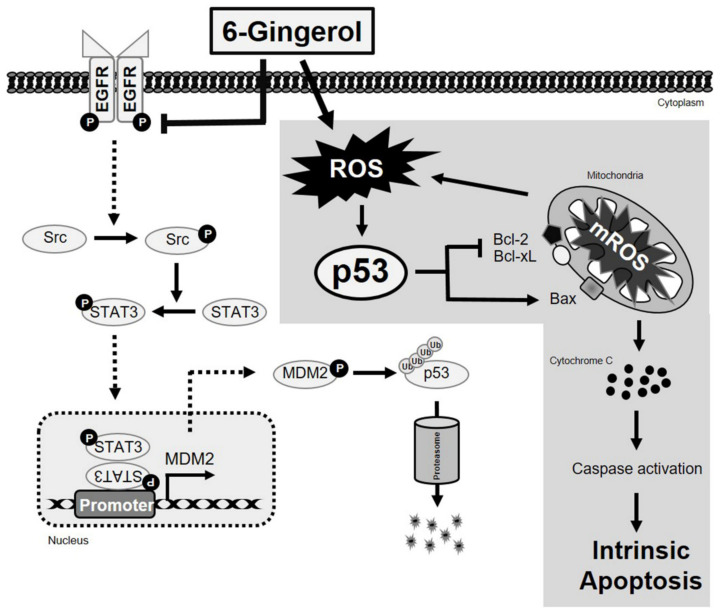
Molecular regulatory mechanism of p53-dependent intrinsic apoptosis by 6-gingerol in breast cancer cells and EGFR/Src/STAT3 signaling pathway inhibition by 6-gingerol.

Our study showed the anti-cancer activity of 6-gingerol by inducing ROS, DDR, cell cycle arrest, and mitochondrial intrinsic apoptosis. Hence, we checked whether 6-gingerol has the ability to inhibit cancer stemness or tumorsphere. In order to analyze the role p53 in tumorsphere formation and its inhibition by 6-gingerol, we cultured MDA-MB-231 and MCF-7 cells in tumorsphere media (DMEM/F12 media containing EGF, bFGF, and B27) with or without 6-gingerol and p the expression of phospho-MDM2 expressions 53 inhibitor, PFT-α and allowed 14 days for sphere formation. Images were captured using microscope in 0, 7, and 14 days, and obtained results demonstrated a similar sphere formation in non-treated control and p53 inhibitor treated cells which significantly reduced by 6-gingerol (Figure 8A). To confirm the tumorsphere formation, we isolated RNA from sphere and analyzed cancer stem cell markers, SOX2, OCT4, and NANOG using real-time PCR. Results showed an increased expression in control and PFT-α which suggested the efficiency of tumor sphere formation and a downregulation of these genes observed in spheres treated with 6-gingerol (Figure 8B). We then analyzed the intrinsic apoptotic factors expression in tumorsphere. We observed an up-regulation in p53 and BAX mRNA level and downregulation in BCL2 mRNA level by treatment with 6-gingerol (Figure 8C). These results also suggested the ability of 6-gingerol to induce intrinsic apoptosis even in tumorsphere which gave a strong background to account for its action against cancer stem cells. In tumorigenesis condition, phosphorylated STAT3 bind to MDM2 gene promoter and activates MDM2 gene which leads to p53 ubiquitination and thereby proteasome degradation. However, 6-gingerol inhibited activation of STAT3 and MDM2 which then resulted in p53 activation to promote intrinsic pathway of apoptosis (Figure 9). Hence, our results proved that 6-gingerol induced p53-dependent intrinsic apoptosis and inhibited cancer stemness through EGFR/Src/STAT3 signaling in breast cancer cells.

## 3. Discussion

Anticancer treatments using conventional therapies are often lacking a specific target as they act by mass killing cells, which can lead to severe side effects. However, treatment of hormone-specific breast cancer cells is also difficult as specific drugs for these hormone-dependent breast cancers exert significant adverse effects. These specific chemotherapeutic drugs kill cancer cells by targeting hormones such as estrogen receptor (ER), progesterone receptor (PR), or human epidermal growth factor receptor 2 (HER2). For example, tamoxifen is identified as chemotherapeutic drug that targets ER-positive cancer cells but produces side effects such as increased tumor or bone pain [6]. Natural compounds are perceived as the best option to increase the effectivity of anticancer treatment with minimal or no side effects. Natural compounds can act alone by targeting oncogenes, or they can synergize the effects of other chemotherapeutic drugs when they are used as a combined treatment [42,43].

Plant products have been traditionally used for disease treatment, and in current clinical trials, more than 50% of drugs are from natural sources [44]. For example, 6-Gingerol is a major active phenolic compound from ginger that possesses anticancer activities. In gastric cancer cells, 6-gingerol increases sensitivity to radiotherapy through inducing cell cycle arrest and apoptosis [45] and enhancing sensitivity to cisplatin by regulating the PI3K/AKT/mTOR pathway [46,47]; anticancer activity of 6-gingerol has also been demonstrated in human cervical and oral cancer cells via mediation of cell cycle arrest and apoptosis [48]. It also has cytoprotection and chemoprevention functions that correlate with anti-oxidant and anti-inflammatory activities by targeting nuclear factor erythroid 2-related factor 2 (NRF2) as well as nuclear factor kappa B (NF-κB) [49]. However, the mechanism behind 6-gingerol in the induction of cell death in breast cancer remains unknown. Here, we initially described the effect of 6-gingerol on cell viability and cell morphology in MDA-MB-231 and MCF-7 breast cancer cells and found that 6-gingerol induced cell death in breast cancer cells, which was deemed a primary step for an antitumor candidate drug as it does not induce toxicity in normal breast cell MCF-10A. Studies on cell viability of 6-gingerol in normal intestinal epithelial cells (IECs) isolated from mouse colon also did not affect largely on cell number as well as cell morphology [50]. These clearly suggest that 6-gingerol targets only cancer cells.

Resisting cell death is one of the hallmarks of cancer. If a natural compound can subvert this resistance, then that compound may be of value as an anticancer drug. Aberrations in DNA can potentially lead to genomic instability and increase the rate of mutation to produce DNA damage that finally results in tumorigenesis. Induction of iNOS leads to the production of ROS [51] which plays a vital role in DNA damage process and further triggers the DNA damage response mechanism [52]. In our study, 6-gingerol treatment increased the level of cellular and mitochondrial ROS as well as iNOS expression in breast cancer cells, suggesting a possible DDR induction. A higher percentage of ROS production induces iNOS expression, which in turn directs an ATM-mediated DDR mechanism [53]. ATM and ATM kinases often work together during DDR mechanism as they bind directly to the DNA ends that regulate p53 activity. During DDR, ATM activates p53, which then activates CHK1/CHK2 signaling and then BRCA1 to induce genome stability or cell cycle arrest or apoptosis [23]. Our results suggested the induction of DDR by 6-gingerol in breast cancer cells as it activates ATM/p53/CHK/BRCA1 signaling which suggested that ATM may also play a key role in 6-gingerol-induced DDR mechanism in MDA-MB-231 cells. However, these results could be confirmed by comparing them with a commercially available anti-oxidant agent.

The DNA damage triggers DDR mechanism and failure of DNA repair induction results in prolonged cell cycle arrest and apoptosis via p53 activation. Signaling from ATM activates p53, which then activates tumor suppressor genes p21 and p27 that then inhibit CDK proteins and cyclin proteins [8]. 6-Gingerol was also determined to activate p53 proteins in the DDR, which then activated p21 and p27 and inhibited expression of cyclin D1, cyclin E, and CDK4 at both the transcriptional and translational levels and arrested the cell cycle in G0/G1 phase of breast cancer cells. These results indicate potential signaling toward apoptosis induction induced by 6-gingerol. Our results also showed that 6-gingerol can induce early and late apoptosis in breast cancer cells. In the intrinsic pathway or mitochondrial apoptosis, BAX and BCL-2 were identified to play a key role in response to p53 signaling. Anti-apoptotic members of BCL-2 family decide the fate of apoptosis and act as anti-pore factors in mitochondrial apoptosis [54]. BAX, which identified as a pro-apoptotic factor, also helps in pore formation in mitochondrial membranes and determines whether apoptosis can proceed [55]. Increases in the expression of BAX and decreases in the expression of BCL-2 lead to the loss of membrane potential and subsequent pore formation in mitochondrial membrane. Through these pores, cytochrome c from mitochondria is released to the cytosol and activates caspases to initiate apoptosis [56]. In MCF-7 cells, caspase 3 is mutated and plays an executioner role in apoptosis and absence of caspase 3 might influence slower execution of apoptosis and increased necrotic death in MCF-7 as we observed from apoptosis experiments by flow cytometry. Our results showed an increase in BAX and decrease in BCL-2 and BCL-xL expression caused by 6-gingerol treatment; it also demonstrated a reduction in ATP formation as well as loss of mitochondrial membrane potential that aids in the release of cytochrome c from mitochondria to cytosol and consequently activates caspase 9-driven intrinsic mitochondrial apoptosis. We also observed a p53-dependent tumorsphere formation and its inhibition by 6-gingerol treatment. MDM2-p53 interaction also plays a key role in tumorsphere formation in breast cancer cells [57]. 6-gingerol also regulated p53-dependent apoptotic factors in tumorsphere which clearly showing the ability of 6-gingerol to target cancer stem cells.

Sustained proliferation signaling, which is identified as another hallmark of cancer, and EGFR inhibitors can be used to prevent this process. Overexpression of EGFR has been associated with most cancer types, including breast cancer [58]. 6-Gingerol has been determined to bind to the EGF receptor in breast cancer cells and blocked phosphorylation without affecting the amount of total EGFR protein. This suggested that 6-gingerol acts via molecular signaling through EGFR. We then checked the downstream targets correlated with EGFR signaling for tumor progression. Src and STAT3 are considered as a downstream signaling pathway for EGFR signaling [35], and we demonstrated an inhibition in the phosphorylation of both Src and STAT3 without altering the expression of Src and STAT3 proteins. This signaling directs translocation of STAT3 to the nucleus where it binds to the p53 gene promoter in order to initiate transcription processes to block p53-MDM2 interaction, which finally resulted in p53 activation and MDM2 inhibition [39] which we proved using STAT3 siRNA treatment. 6-Gingerol also activated p53 expression, inhibited the phosphorylation of MDM2, and induced apoptosis in p53 siRNA-treated cells by upregulating BAX expression and downregulating BCL-2 expression compared with the results from p53 siRNA-treated cells only. These findings clearly suggest the role of p53 in apoptosis induction by 6-gingerol. Even though MDA-MB-231 cells carry a mutated p53 gene (R280K mutation) and R280K is known to be a gain-of-function mutation [59,60]. 6-gingerol treatment resulted in the increase in the expression of p53 and quicker apoptosis execution via BAX/BCL-2 regulation in MDA-MB-231 than in MCF-7, supporting the hypothesis that p53 in MDA-MB-231 is indeed gain-of-function mutant.

## 4. Materials and Methods

### 4.1. Antibodies and Cell Culture Reagents

Roswell Park Memorial Institute-1640 (RPMI-1640) medium, penicillin-streptomycin solution, and trypsin-EDTA (0.05%) were all purchased from Gibco (Thermo Fisher Scientific, Inc., Waltham, MA, USA). 6-Gingerol (cat no. 23513-14-6) was purchased from TCI (Tokyo Chemical Industry Co., Tokyo, Japan). Fetal bovine serum (FBS) was obtained from Sigma–Aldrich (Merck KGaA, St. Louis, MO, USA). Recombinant human EGF (AF-100-15) was purchased from Peprotech, Inc. (Rocky Hill, NJ, USA). Antibodies specific for CDK4 (sc-23896), BCL-2 (sc-7382), p21 (sc-6246), and cyclin E (sc-377100) together with secondary antibodies (anti-mouse (sc-516102) and anti-rabbit (sc-2357)) were purchased from Santa Cruz Biotechnology (Dallas, TX, USA). Antibodies specific for pEGFR (#3777), EGFR (#4267), pSTAT3 (#9145), STAT3 (#9139), cyclin D1 (#2922), p27 (#3686), p53 (#9282), pCHK1 (#2348), pATM (#5883), pBRCA1 (#9009), pHistone H2A.X (#9718), pCHK2 (#2197), BAX (#2772), Casp 9 (#9502), C-Casp 9 (#9505), cytochrome c (#11940), BCL-xL (#2764), pMDM2 (#3521), MDM2 (#86934), pSrc (#6943), Src (#2109), and GAPDH (#2118) were obtained from Cell Signaling Technology (Beverly, MA, USA). Finally, iNOS antibody (NB300-650) was purchased from Novus Biologicals (Littleton, CO, USA).

### 4.2. Cell Culture and Treatment

MDA-MB-231 (no. 30026, Korean Cell Line Bank, Seoul, Korea) and MCF-7 (no. 30022, Korean Cell Line Bank, Seoul, Korea) cell lines were cultured in RPMI-1640 supplemented with 10% FBS and 1% penicillin and streptomycin at 37 °C in 5% CO_2_. MCF-10A immortalized normal human breast epithelial cells [CRL-10317; American Type Culture Collection (ATCC), Manassas, VA, USA] were grown to confluence in phenol red-free DMEM/F12 medium (11320033; Gibco; Thermo Fisher Scientific, Inc.) supplemented with cholera toxin (1 mg/mL; C8052, Sigma-Aldrich; Merck KGaA), insulin (10 mg/mL; I0516; Sigma-Aldrich; Merck KGaA), EGF (5 mg/mL; E9644; Sigma-Aldrich; Merck KGaA), 1% penicillin/streptomycin and 5% FBS. Cells were grown to 80% confluence; then, they were gently washed twice with phosphate-buffered saline (PBS). Unless otherwise specified, the cells were treated with various concentrations of 6-gingerol at 37 °C for different time periods according to the experiment pattern.

### 4.3. Cell Proliferation Inhibition

Cell viability was assayed by MTT assay. Briefly, cells were re-suspended in RPMI-1640 one day before drug treatment, at a density of 3 × 10^3^ cells per well in 96-well culture plates. In non-treated control cells, culture medium was replaced with fresh medium containing dimethyl sulfoxide (DMSO) as a vehicle control. For treatment, cells were incubated with increasing concentrations of 6-gingerol (from 10 to 500 μM). Following drug treatment, MTT (5 mg/mL) was added, with culture dishes incubated for 4 h at 37 °C. The resulting formazan product was dissolved in DMSO, and the absorbance was read at 560 nm on an Ultra Multifunctional Microplate Reader (Tecan, Durham, NC, USA). All measurements were performed in triplicate, with experiments repeated at least three times.

### 4.4. DAPI Staining and Morphological Analysis

Apoptotic condensed chromatin was examined by DAPI staining. MDA-MB-231 and MCF-7 cells were seeded in 6-well plates at a density of 1.5 × 10^5^ cells/well and were treated with different concentrations of 6-gingerol for 48 h, after which the cells were washed twice with PBS. Then, 500 μL of 300 nM DAPI staining solution was added, and the cells were washed twice with PBS. Stained cells were mounted using mounting solution on microscope slides, wherein they were observed by fluorescence microscopy (Olympus IX71/DP72, Tokyo, Japan).

### 4.5. Western Blotting

Whole cell lysates were prepared by incubating untreated or 6-gingerol-treated MDA-MB-231 and MCF-7 cells on ice with radioimmunoprecipitation lysis buffer (20-188; EMD Millipore, Burlington, MA, USA) containing phosphatase and protease inhibitors. Protein concentrations were measured via the Bradford method (Thermo Fisher Scientific, Inc., Waltham, MA, USA). Equal amounts of protein (100 μg/well) were resolved with 10% SDS-PAGE. Separated proteins were then transferred onto nitrocellulose membranes. The blots were blocked for 1 h with 5% skim milk (BD Biosciences, San Jose, CA, USA) in TBS-T buffer (20 mM Tris-HCl (Sigma–Aldrich; Merck KGaA, St. Louis, MO, USA), pH 7.6, 137 mM NaCl (Formedium, Norfolk, UK; NAC03), 0.1× Tween 20 (Scientific Sales, Inc. Oak Ridge, TN, USA). The membranes were then incubated overnight at 4 °C in a shaker with primary antibodies diluted in 5% bovine serum albumin (EMD Millipore). The membranes were then washed with TBS-T and incubated for 1 h at room temperature with HRP-conjugated secondary antibodies. Detection was performed using a Femto Clean Enhanced Chemiluminescence Solution Kit (GenDEPOT; 77449; Katy, TX, USA) and a LAS-4000 imaging device (Fujifilm, Tokyo, Japan).

### 4.6. Reverse Transcriptase-Quantitative Polymerase Chain Reaction (RT-qPCR)

Total RNA was extracted using the RNeasy Mini Kit (Qiagen GmbH, Hilden, Germany). The RNA was quantified spectrophotometrically at 260 nm, and cDNA was synthesized from the total RNA at 42 °C for 1 h and at 95 °C for 5 min using a first-strand cDNA synthesis kit (Bioneer Corporation, Daejeon, Korea) and oligo d(T) primers. The RT-PCR Premix Kit (Bioneer Corporation) was used to amplify CCND1, CCNE1, CDK4, CDKN1A, CDKN1B, BAX, BCL-2, CYCS, CASP9, and GAPDH cDNA with primers synthesized by the Bioneer Corporation. qPCR was conducted in a thermal cycler (C1000 Thermal Cycler, Bio-Rad, Hercules, CA, USA) as follows: 2 μL of diluted cDNA was mixed with forward and reverse primers (1 μL each, 100 pM) and 10 μL of TB Green Advantage Premix (Takara Bio, Shiga, Japan). qPCR conditions were as follows: initial denaturation at 95 °C for 5 min, followed by 40 cycles of denaturation at 95 °C for 40 s, annealing at 58 °C for 40 s, extension at 72 °C for 40 s, and a final extension at 72 °C for 5 min. The primers used for the amplification are listed in Table S1. All measurements were performed in triplicate. The relative expression of the target genes was normalized to GAPDH. The calculations were carried out using the Cp values.

### 4.7. Cell Cycle Analysis

The DNA content of the 6-gingerol-treated and non-treated cells was determined using a BD Cycletest Plus DNA Reagent Kit (BD Biosciences, San Jose, CA, USA). Approximately 5 × 10^5^ cells, treated with or without 6-gingerol for 48 h, were washed with PBS and permeabilized with trypsin. RNA interaction with PI was neutralized by treating the cells with RNase buffer and trypsin inhibitor. The samples were then stained with PI and incubated for 30 min in the dark at room temperature and analyzed using a FACSCalibur flow cytometer (BD Biosciences, San Jose, CA, USA).

### 4.8. Apoptosis Analysis

Annexin V-FITC was used to measure the apoptosis in MDA-MB-231 and MCF-7 cells. 6-Gingerol-treated cells were washed with PBS, re-suspended in binding buffer to a concentration of 1 × 10^6^ cells. The cells were stained with annexin V-FITC and PI for 10 min in the dark at room temperature. The percentage of apoptotic cells was measured by flow cytometry via FACSCalibur and was analyzed using FlowJo software.

### 4.9. FACS Analysis for Mitochondrial Membrane Potential and ROS

After the cultured cells were harvested and washed with 1 mL of pre-warmed no-glucose RPMI-1640 medium (Gibco) supplemented with 10% FBS (staining buffer), 1 × 10^6^ cells were re-suspended in 1 mL of staining buffer containing MitoTracker Deep Red (40 nM; Invitrogen, Carlsbad, CA, USA: M22426) for mitochondrial membrane potential, CM-H2DCFDA (5 μM; Invitrogen, C6827) for cellular ROS, and MitoSOX (5 μM; Invitrogen, M36008) for mitochondrial ROS. Cells were then placed in a CO_2_ incubator at 37 °C for 30 min. The stained cells were washed with 1 mL of pre-warmed staining buffer and were used for flow cytometry analysis.

### 4.10. Comet Assay

The comet assay kit (Abcam, Cambridge, MA, USA) was used for measuring cellular DNA damage. This assay is a single-cell gel electrophoresis method for a simple evaluation of cellular DNA damage. A base layer of comet agarose was created on a slide, and then, a layer of cells and agarose was added, followed by lysis. Electrophoresis was performed under neutral conditions, and the cells were stained with DNA dye. Cell morphology was observed by fluorescence microscopy (Olympus IX71/DP72).

### 4.11. ATP Determination Assay

The ATP Determination Kit (Molecular Probes, Eugene, OR, USA) was used for measuring ATP. Briefly, MDA-MB-231 and MCF-7 cells were treated with 6-gingerol, and an equal amount of cells was collected for the ATP determination assay. The standard reaction solution for the samples was made using reaction buffer, DTT, D-luciferin, and firefly luciferase as provided in the kit; then, cells were added along with the standard reaction solution. Luminescence readings were taken immediately using a plate-reading luminometer, and calculations were done according to assay protocol.

### 4.12. Isolation of Mitochondria/Cytosol Fractions

Mitochondria/cytosol fractions from 6-gingerol-treated and non-treated MDA-MB-231 and MCF-7 cells were extracted using a mitochondria/cytosol fractionation kit (Abcam). 1× cytosol extraction buffer containing DTT and protease inhibitors was added to the cells (5 × 10^7^), which were then incubated on ice for 10 min. After incubation, samples were centrifuged at 700× *g* for 10 min at 4 °C to collect the supernatant, which was centrifuged again at 10,000× *g* for 30 min at 4 °C. The supernatant was removed for testing of the cytosol fraction, while the pellet was re-suspended in PBS to obtain the mitochondrial fraction; Western blotting of cytochrome c was carried out as described above.

### 4.13. siRNA Transfection

MDA-MB-231 and MCF-7 cells (1 × 10^6^ cells) were seeded in 6-well plates and grown to 60% confluence and then transfected with STAT3 siRNA (sc-29493) or p53 siRNA (sc-29435; Santa Cruz Biotechnology, Dallas, TX, USA) using Lipofectamine Transfection Reagent (Thermo Fisher Scientific, Inc., Waltham, MA, USA). After 24 h, transfected cells were additionally cultured with or without 6-gingerol for another 48 h under cell culture conditions.

### 4.14. Tumor Sphere Formation Assay

Tumor sphere assay was performed by culturing MDA-MB-231 and MCF-7 cells (5 × 10^3^ cells/well) in DMEM/F12 media (11330-032; Gibco, Thermo Fisher Scientific, Inc., Waltham, MA, USA) containing growth supplements including EGF (324831; Sigma-Aldrich), bFGF (F0291; Sigma-Aldrich) and B27 (17504-044: Gibco) in low attachment 6-well plates. The treatment started at day 0 and incubation continued for 14 days. Photographs were taken at day 0, 7, and 14 using a microscope. Total RNA was isolated from the spheres on 14th day and analyzed using real time PCR. Tumor sphere number was counted using microscope.

### 4.15. Statistical Analyses

All experiments were performed at least three times. The results were expressed as the mean ± standard error of the mean. Statistical analyses were conducted via one-way analysis of variance (ANOVA) or Student’s *t*-test. The one-way ANOVA was performed with Tukey’s post-hoc test. The analyses were performed using SAS 9.3 software program (SAS Institute, Inc., Cary, NC, USA). A *p*-value < 0.05 (*) was considered to indicate a significant difference.

## 5. Conclusions

In this study, we have determined that 6-gingerol can induce ROS production, DDR, cell cycle arrest, mitochondrial apoptosis and can inhibit cancer stemness in MDA-MB-231 and MCF-7 breast cancer cells. Furthermore, 6-gingerol was determined to activate the p53 expression via EGFR/Src/STAT3 signaling, thereby inducing p53-dependent intrinsic apoptosis in breast cancer cells. Altogether, 6-gingerol can be considered as a candidate drug for the treatment of hormone-specific breast cancer cells.

## Data Availability

Data available on request due to privacy.

## Supplementary Materials

The following are available online at https://www.mdpi.com/article/10.3390/ijms22094660/s1.
