# Supplementary material for: Potential Antitumor Effects of 6-Gingerol in p53-Dependent Mitochondrial Apoptosis and Inhibition of Tumor Sphere Formation in Breast Cancer Cells
Source: Int J Mol Sci. 2021 Apr 28;22(9):4660. doi: 10.3390/ijms22094660 (PMC8124719; doi:10.3390/ijms22094660)
Supplement: Supplementary file 1 [file ijms-22-04660-s001.zip › Supplementary Files/Table S1.docx]

| **Sl No** | **Gene** | **Annealing temperature (^o^C)** | **Sequence (5` - 3`)** |
| --- | --- | --- | --- |
| 1 | *CCND1* | 58 | F: 5’-tcaccctgagagtagggagc-3’  R: 5’-gttaagaggtctgcccaccc-3’ |
| 2 | *CCNE1* | 58 | F: 5’-agcagcaccccatgacac-3’  R: 5’-catgtggcctgcagctct-3’ |
| 3 | *CDK4* | 58 | \| F: 5’-gcgagatggacagatccctg-3’ \| \| --- \| \| R: 5’-ctcgtaaggagaggtgggga-3’ \| |
| 4 | *CDKN1A* | 58 | F: 5’-ggtggctgaaggcttcgt-3’  R: 5’-ttgtggcgcgattctggt-3’ |
| 5 | *CDKN1B* | 58 | F: 5’-aagcgagtcagcgcaagt-3’  R: 5’-gatgcgttcggctacgga-3’ |
| 6 | *BAX* | 58 | F: 5’-atgcgtccaccaagaagc-3’  R: 5’-cagctgccactcggaaaa-3’ |
| 7 | *BCL-2* | 58 | F: 5’-tggggtcatgtgtgtgga-3’  R: 5’-cccagcctccgttatcct-3’ |
| 8 | *CYCS* | 58 | F: 5’-cccaagcacttctggtgg-3’  R: 5’-atcacgccattgcactcc-3’ |
| 9 | *CASP9* | 58 | F: 5’-ggacatgctggcttcgtt-3’  R: 5’-tgggtgtttccggtctga-3’ |
| 10 | *GAPDH* | 58 | F: 5’-cccactcctccacctttgac-3’  R: 5’-tcctcttgtgctcttgctgg-3’ |

**Table S1. q-PCR primer sequences, annealing temperature and product sizes.**
